# Real‐world patient characteristics, treatment patterns, and treatment outcomes of patients with diffuse large B‐cell lymphoma by line of therapy

**DOI:** 10.1002/cam4.7173

**Published:** 2024-04-10

**Authors:** Helmneh M. Sineshaw, Christina M. Zettler, Jennifer Prescott, Mahek Garg, Samhita Chakraborty, Eric M. Sarpong, Claire Bai, Andrew J. Belli, Laura L. Fernandes, Ching‐Kun Wang

**Affiliations:** ^1^ Merck & Co., Inc. Rahway New Jersey USA; ^2^ COTA, Inc. New York New York USA

**Keywords:** DLBCL, outcomes, relapsed/refractory, survival, treatment

## Abstract

**Background:**

Although initial treatment of diffuse large B‐cell lymphoma (DLBCL) with rituximab, cyclophosphamide, doxorubicin, vincristine, and prednisone (R‐CHOP) can be effective, up to 50% of patients will develop refractory or relapsed (R/R) disease. This study aimed to provide contemporary data on characteristics, treatment patterns, and outcomes for R/R‐DLBCL.

**Methods:**

Patients with incident (January 2016 to March 2021) DLBCL age ≥18 years who initiated first‐line (1L) therapy were identified from the COTA real‐world database. Baseline characteristics, treatment patterns, and real‐world outcomes, including time to next treatment (rwTTNT) and overall survival (rwOS), were assessed for the study population and by line of therapy (LOT).

**Results:**

A total of 1347 eligible DLBCL patients were identified. Of these, 340 (25.2%) proceeded to receive 2L, of whom 141 (41.5%) proceeded to receive 3L, of whom 51 (36.2%) proceeded to receive 4L+. Most common treatments were R‐CHOP in 1L (63.6%), stem cell transplant (SCT) in 2L (17.9%), polatuzumab vedotin, bendamustine, and rituximab (Pola‐BR) in 3L (9.9%), and chimeric antigen receptor T‐cell therapy (CAR‐T) in 4L (11.8%). Treatment patterns were more variable in later LOTs. One‐ and 3‐year rwOS from 1L initiation were 88.5% and 78.4%, respectively. Patients who received later LOTs experienced numerically lower 1‐ and 3‐year rwOS (from 2L initiation: 62.4% and 46.4%, respectively).

**Conclusions:**

In this real‐world analysis, 25.2% of patients experienced R/R‐DLBCL after 1L with poor outcomes. Given the findings of this study, there is a high unmet need for novel, safe, and effective treatment options for patients with R/R DLBCL.

## Graphical abstract

More than a quarter of real‐world patients with DLBCL experience R/R disease following 1L treatment, and outcomes for patients with R/R disease are poor. There is a high unmet need for novel, safe, and effective treatment options for patients with R/R DLBCL.

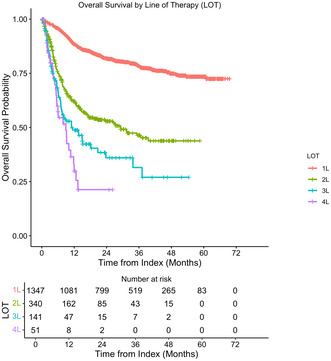

## 1 INTRODUCTION

Non‐Hodgkin lymphoma (NHL) is the most common hematologic malignancy with over 80,500 estimated new cases diagnosed in the United States in 2023.^1^ Diffuse large B‐cell lymphoma (DLBCL) is the most frequent subtype of NHL, accounting for 30%–40% of cases.^2^ DLBCL is an aggressive malignancy with heterogenous biology and behavior. A variety of patient and clinical characteristics (e.g., age, stage, and tumor bulk), prognostic indices (e.g., International Prognostic Index (IPI) score) and gene expression profiling are used for disease risk stratification and treatment planning.^3^

First‐line (1L) standard of care (SOC) for DLBCL remains chemoimmunotherapy with R‐CHOP (rituximab, cyclophosphamide, doxorubicin, vincristine, and prednisone). Although nearly 60% of patients achieve complete remission to 1L treatment, up to 50% could develop refractory or relapsed (R/R) disease, which carries a poor prognosis.^4^, ^5^ Though high‐dose chemotherapy followed by autologous stem cell transplant (ASCT) is the recommended SOC for eligible patients in the 2L setting based on results from the pivotal PARMA study, real‐world SOC in this setting remains less clearly defined.^6^, ^7^

The emergence of novel treatment strategies, such as chimeric antigen receptor T‐cell (CAR‐T) therapy in October 2017, polatuzumab in June 2019, and targeted agents, has provided additional treatment options for patients with R/R DLBCL, but their real‐world uptake and outcomes remain poorly understood and quantified.^8^, ^9^ As such, continuous evaluation of patients with DLBCL treated in the real‐world setting in the contemporary treatment landscape is needed. This study sought to address this need by examining the demographic and clinical characteristics, treatment patterns, and real‐world time to next treatment (rwTTNT) and overall survival (rwOS) of patients with DLBCL by line of therapy (LOT) to provide contemporary data and identify real‐world unmet need in line with the new treatment advances.

## 2 METHODS

### 2.1 Study population

This retrospective, observational cohort study utilized the COTA, Inc. real‐world database, a Health Insurance Portability and Accountability Act (HIPAA)‐compliant database comprised of longitudinal data abstracted from electronic health records (EHR) pertaining to the diagnosis, clinical management, and outcomes of patients with cancer. COTA's data abstraction process leverages both human abstraction and technologic methods to transform structured and unstructured data into a standardized data model. The data are subject to extensive quality assurance processes including assessments for missingness, distribution, and clinical plausibility of data entries. Data are abstracted from the EHR of COTA's partnered healthcare provider sites, which include both academic centers and community networks with geographic concentration in the Northeast and Southern regions of the United States.

**FIGURE 1:**
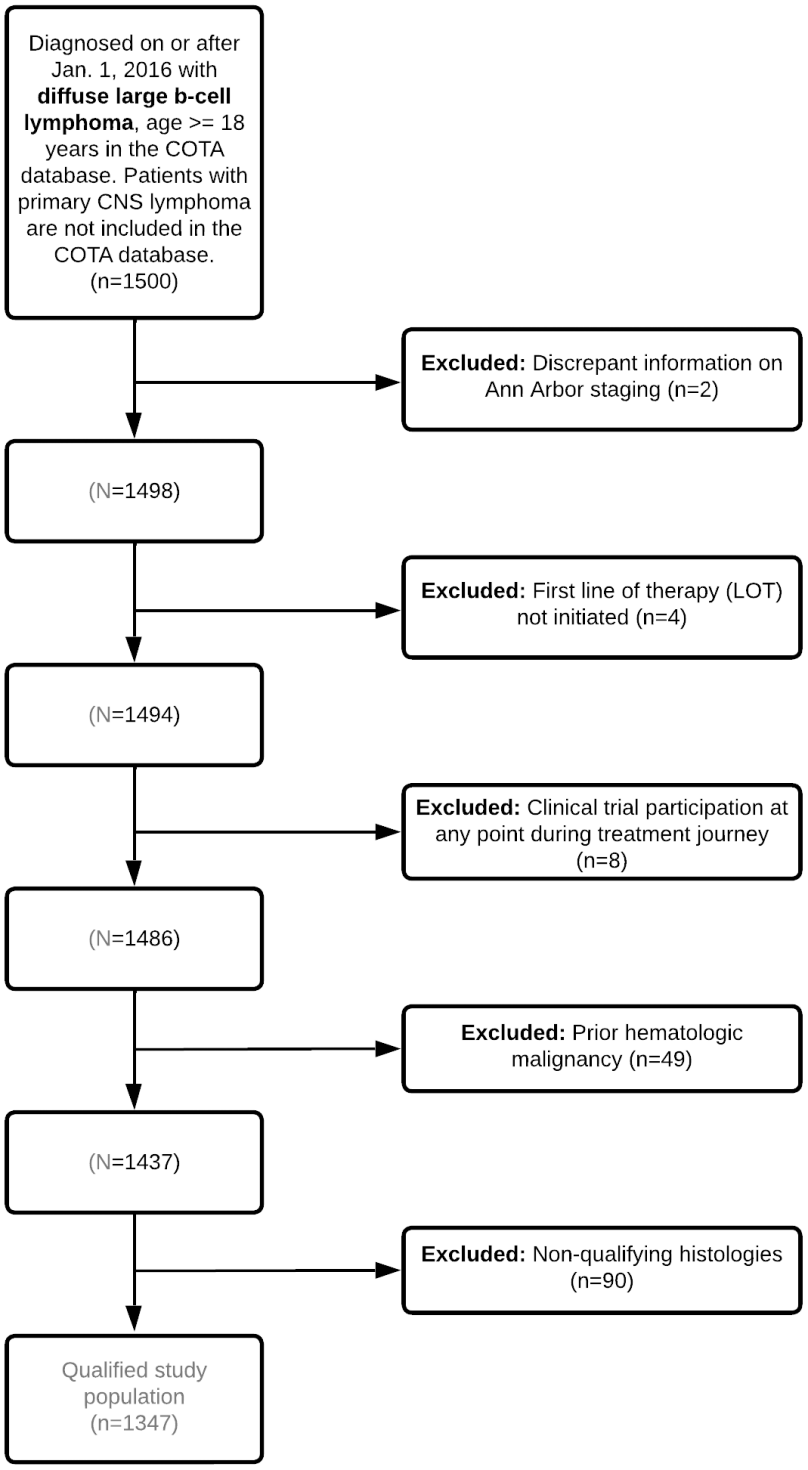
Patient attrition diagram.

The initial study population included a sample of 1500 adult patients (age ≥18 years) with incident (newly diagnosed), clinician‐ and/or pathology‐confirmed, non‐primary central nervous system (CNS) DLBCL on or after January 1, 2016 until March 31, 2021. Patients with primary CNS DLBCL were not included due to the differences in the treatment paradigm between this population and other patients with DLBCL. From the initial sample, the following additional criteria were applied: available Ann Arbor stage (I–IV) documentation (due to its importance as a prognostic factor), initiated 1L treatment, diagnosis with one of the following histologies: DLBCL, NOS, intravascular large B‐cell lymphoma, or T‐cell histiocyte‐rich large B‐cell lymphoma, no clinical trial participation in treatment journey, and no prior diagnosis of a hematologic malignancy documented by the treating clinician in the EHR (Figure 1). Data for this cohort are available from the date of patient diagnosis between January 1, 2016 and March 31, 2021 (inclusive) and a cutoff date for follow‐up of December 8, 2021. Diagnosis after January 1, 2016 was chosen to identify patients treated in the most contemporary treatment setting following the uptake of R‐CHOP as 1L standard of care, and March 31, 2021 was chosen to allow for sufficient follow‐up time for response and progression assessment following diagnosis and prior to the December 8, 2021 data cutoff date.

Major demographic variables included age, sex, race, ethnicity, region, and practice setting (academic vs. community). Existing comorbidities present at the time of diagnosis were classified using the Charlson Comorbidity Index (CCI).^10^ Clinical variables included year of diagnosis, DLBCL subtype, Ann Arbor stage, assessment of molecular markers of interest (classified as present, absent, or unknown), B symptoms, and bulky tumor, among other variables. National Comprehensive Cancer Network (NCCN) International Prognostic Index (IPI) score was calculated using available data elements, and missingness in any of the variables used to calculate the score resulted in a missing NCCN IPI Score.^11^ Treatment regimens received and reasons for premature discontinuation (i.e., physician‐documented reason for discontinuation prior to intended completion) were also summarized. Reasons for discontinuation included death, doctor preference, drug shortage, inadequate response, insurance reasons, pandemic/epidemic, patient preference, progression, toxicity, and unknown due to missing documentation.

Index dates for the study were defined as the start of each LOT for DLBCL. Window for assessment of baseline patient characteristics was the baseline period: any point prior to and until the date of 1L therapy initiation. Clinical characteristics that vary over time, such as ECOG status, NCCN IPI score, and molecular markers, were reassessed at the start of each LOT, if available. If not available, results from prior LOT were used. The follow‐up period, defined as the duration of time from the appropriate index date to the date of patient death, or last visit date (if date of death not available) before December 8, 2021, was used to assess relevant study variables and outcomes.

LOT was assigned programmatically to the abstracted data using a disease‐specific algorithm developed by COTA using real‐world data best practices and relevant industry and clinical guidelines. The algorithm was reviewed and vetted by external oncologists. The algorithm begins with the first regimen received for the cancer diagnosis, and all regimens received within a specified timeframe of the initial regimen are combined into a single LOT, absent any documented progression. LOT will continue until a regimen is discontinued due to progression or inadequate response, a new drug or regimen is added outside of the prespecified window, a gap in treatment occurs, or the patient undergoes CAR‐T cell therapy, all of which will end the current LOT and initiate a new line. Conditioning regimens, maintenance regimens, SCTs within 1 year of preceding therapy, and the de‐escalation or dropping of drugs from a regimen will not advance the LOT. Refractory disease was defined as MD‐stated advancement of disease not following a complete response within or following a given LOT, but prior to the subsequent LOT. Disease relapse was defined as MD‐stated advancement of disease following a complete response within or following a given LOT, but prior to the subsequent LOT.

### 2.2 Study outcomes and statistical analyses

Descriptive analysis was conducted to summarize patient characteristics for the study population and by LOT. Continuous variables were described by medians and interquartile ranges. Categorical variables were described by patient counts and percentages. There was no formal hypothesis testing in this study, and all analyses were descriptive in nature.

rwOS and rwTTNT were summarized by LOT as the duration of time from index date until date of death (or initiation of next LOT for rwTTNT) using the Kaplan–Meier (KM) method. Patients were censored at date of last visit if event date was not available. KM estimates of rwOS and rwTTNT and Greenwood 95% confidence intervals were calculated for the study population and by LOT, and KM curves by LOT were generated. Follow‐up began at the time of initiation of a given LOT until event or censor. One‐, 3‐, and 5‐year rwOS rates were summarized by LOT as the proportion of patients who did not experience a death event within the relevant time frame among all patients who initiated that LOT.

All statistical analyses were performed using R Statistical Software (v4.2.2; R Core Team 2022). COTA's de‐identified real‐world database is approved by WCG IRB for use in research (Study #1174746).

## 3 RESULTS

A total of 1347 patients were identified in the COTA database meeting the study criteria. Of the 1347 patients that received 1L therapy, 340 (25.2%; *N* = 340/1347) subsequently received 2L therapy. Of the 340 2L patients, 141 (41.5%; *N* = 141/340) received 3L therapy, of whom 51 (36.2%; *N* = 51/141) received 4L therapy or later LOTs (Table 1). Among patients receiving 1L therapy, the median age at diagnosis was 67 years, and the majority of patients were male (54.9%) and treated in a community practice (81.3%). More than 38% of patients were diagnosed with Stage IV disease. The proportion of patients with Stage I disease at diagnosis was numerically lower with each subsequent LOT.

**TABLE 1:** Patient and clinical characteristics.

	All/1L *N* = 1347	2L *N* = 340	3L *N* = 141	4L *N* = 51
Age at initial Dx, median [IQR]	67.0 [57.0, 75.0]	67.0 [56.8, 74.0]	66.0 [55.0, 72.0]	63.0 [54.5, 71.5]
Sex, *n* (%)				
Female	607 (45.1)	139 (40.9)	65 (46.1)	22 (43.1)
Male	740 (54.9)	201 (59.1)	76 (53.9)	29 (56.9)
Race, *n* (%)				
Asian	47 (3.5)	20 (5.9)	9 (6.4)	4 (7.8)
Black or African American	68 (5.0)	23 (6.8)	10 (7.1)	3 (5.9)
White	1070 (79.4)	255 (75.0)	103 (73.0)	35 (68.6)
Other	128 (9.5)	34 (10.0)	13 (9.2)	5 (9.8)
Unknown	34 (2.5)	8 (2.4)	6 (4.3)	4 (7.8)
Ethnicity, *n* (%)				
Hispanic	284 (21.1)	74 (21.8)	35 (24.8)	16 (31.4)
Non‐Hispanic	708 (52.6)	173 (50.9)	69 (48.9)	21 (41.2)
Unknown	355 (26.4)	93 (27.4)	37 (26.2)	14 (27.5)
Practice setting, *n* (%)				
Academic	252 (18.7)	68 (20.0)	32 (22.7)	14 (27.5)
Community	1095 (81.3)	272 (80.0)	109 (77.3)	37 (72.5)
Year of diagnosis, *n* (%)				
2016	331 (24.6)	88 (25.9)	38 (27.0)	11 (21.6)
2017	314 (23.3)	86 (25.3)	30 (21.3)	11 (21.6)
2018	310 (23.0)	77 (22.6)	35 (24.8)	17 (33.3)
2019	259 (19.2)	65 (19.1)	33 (23.4)	12 (23.5)
2020	118 (8.8)	22 (6.5)	4 (2.8)	0 (0.0)
2021	15 (1.1)	2 (0.6)	1 (0.7)	0 (0.0)
Follow‐up time from diagnosis in months, median [IQR]	28.0 [14.6, 43.0]	22.2 [12.5, 37.6]	22.8 [14.0, 38.2]	23.7 [16.3, 36.0]
Time from diagnosis to initiation of the given LOT in months, median [IQR]	0.8 [0.5, 1.3]	8.1 [5.5, 13.3]	12.5 [8.6, 21.6]	17.2 [10.9, 26.9]
Follow‐up time from index in months, median [IQR]	26.7 [13.6, 41.9]	10.5 [4.2, 23.1]	6.7 [2.7, 14.6]	5.5 [2.0, 10.4]
ECOG status, *n* (%)				
0–1	878 (65.2)	195 (57.4)	80 (56.7)	28 (54.9)
≥2	192 (14.3)	54 (15.9)	20 (14.2)	8 (15.7)
Unknown	277 (20.6)	91 (26.8)	41 (29.1)	15 (29.4)
DLBCL subtype, *n* (%)				
Activated B‐cell–like	434 (32.2)	110 (32.4)	38 (27.0)	17 (33.3)
Activated B‐cell–like and germinal center B‐cell–like	6 (0.4)	4 (1.2)	1 (0.7)	0 (0.0)
Germinal center B‐cell–like	530 (39.3)	110 (32.4)	55 (39.0)	14 (27.5)
Unknown	377 (28.0)	116 (34.1)	47 (33.3)	20 (39.2)
Ann Arbor stage, *n* (%)				
1	172 (12.8)	19 (5.6)	4 (2.8)	0 (0.0)
2	313 (23.2)	55 (16.2)	27 (19.1)	12 (23.5)
3	342 (25.4)	96 (28.2)	33 (23.4)	17 (33.3)
4	520 (38.6)	170 (50.0)	77 (54.6)	22 (43.1)
NCCN International Prognostic Index (IPI), *n* (%)				
Low risk (Score 0–1)	82 (6.1)	12 (3.5)	3 (2.1)	2 (3.9)
Low‐intermediate risk (Score 2–3)	340 (25.2)	73 (21.5)	28 (19.9)	10 (19.6)
High‐intermediate risk (Score 4–5)	361 (26.8)	79 (23.2)	32 (22.7)	13 (25.5)
High risk (Score 6+)	95 (7.1)	34 (10.0)	16 (11.3)	4 (7.8)
Unknown	469 (34.8)	142 (41.8)	62 (44.0)	22 (43.1)
Baseline CCI score, *n* (%)				
0	497 (36.9)	110 (32.4)	44 (31.2)	17 (33.3)
1–2	533 (39.6)	137 (40.3)	60 (42.6)	24 (47.1)
3–4	216 (16.0)	65 (19.1)	29 (20.6)	8 (15.7)
5+	101 (7.5)	28 (8.2)	8 (5.7)	2 (3.9)
CCI comorbidity present, *n* (%)				
Diabetes without chronic complications	326 (24.2)	88 (25.9)	36 (25.5)	9 (17.6)
Renal disease	190 (14.1)	64 (18.8)	29 (20.6)	10 (19.6)
Chronic pulmonary disease	166 (12.3)	44 (12.9)	20 (14.2)	7 (13.7)
Molecular markers				
BCL2, *n* (%)				
Negative	236 (17.5)	35 (10.3)	9 (6.4)	5 (9.8)
Positive	947 (70.3)	274 (80.6)	120 (85.1)	40 (78.4)
Unknown	164 (12.2)	31 (9.1)	12 (8.5)	6 (11.8)
BCL6, *n* (%)				
Negative	141 (10.5)	40 (11.8)	21 (14.9)	7 (13.7)
Positive	1062 (78.8)	272 (80.0)	111 (78.7)	40 (78.4)
Unknown	144 (10.7)	28 (8.2)	9 (6.4)	4 (7.8)
C‐MYC, *n* (%)				
Negative	456 (33.9)	108 (31.8)	51 (36.2)	16 (31.4)
Positive	544 (40.4)	168 (49.4)	69 (48.9)	24 (47.1)
Unknown	347 (25.8)	64 (18.8)	21 (14.9)	11 (21.6)
CD10, *n* (%)				
Negative	590 (43.8)	155 (45.6)	53 (37.6)	25 (49.0)
Positive	611 (45.4)	151 (44.4)	71 (50.4)	21 (41.2)
Unknown	146 (10.8)	34 (10.0)	17 (12.1)	5 (9.8)
CD20, *n* (%)				
Negative	14 (1.0)	5 (1.5)	3 (2.1)	2 (3.9)
Positive	1229 (91.2)	315 (92.6)	127 (90.1)	46 (90.2)
Unknown	104 (7.7)	20 (5.9)	11 (7.8)	3 (5.9)

Among 1L treated patients, 340 (25.2%) had low‐intermediate risk and 361 (26.8%) had high‐intermediate risk disease based on their NCCN IPI score. About one‐third of the 1L population had a missing NCCN IPI score. In 1L, 32.2% (*N* = 434/1347) of patients had activated B‐cell–like (ABC) DLBCL, and 39.3% (*N* = 530/1347) of patients had germinal center B‐cell–like (GCB) DLBCL. The proportions of patients with each subtype were broadly consistent across LOTs. The proportion of patients among the 1L treated population that tested positive for some of the major molecular markers were: 91.2% for CD20, 78.8% for BCL6, 70.3% for BCL2, 45.4% for CD10, and 40.4% for C‐MYC. Diabetes without chronic complications (24.2% in 1L), renal disease (14.1% in 1L), and chronic pulmonary disease (12.3% in 1L) were the three most common comorbidities. About one‐quarter (23.5%) of the study population had a baseline CCI comorbidity score of 3 or more.

Across 1L, 2L, 3L, and 4L, 16.0%, 41.8%, 39.0%, and 56.9% of patients prematurely discontinued therapy respectively. **(**Table 2) Among all patients receiving 1L, 12.8% (*N* = 172/1347) experienced refractory disease and 11.7% (*N* = 157/1347) experienced disease relapse. After 1L therapy, 23% (*N* = 310/1347) of patients had a documented progression/relapse event in the EHR within 12 months, and 1.4% (*N* = 19/1347) of patients experienced progressive disease more than 12 months after 1L initiation and prior to 2L initiation. Nearly 49% (*N* = 166/340) of 2L patients, 40.5% (*N* = 57/141) of 3L patients, and 54.9% (*N* = 28/51) of 4L patients experienced refractory disease or disease relapse. Of patients who received 3 L therapy, 7.1% (*N* = 10/141) had prior exposure to SCT and 3.5% (*N* = 5/141) had prior exposure to polatuzumab. There was prior exposure to SCT in 15.7% (*N* = 8/51) and prior exposure to polatuzumab in 21.6% (*N* = 11/51) of patients who received 4L of therapy.

**TABLE 2:** Treatment discontinuation, refractory disease, and disease relapse by line of therapy.

	Line 1	Line 2	Line 3	Line 4
Number treated, *n* (%)	1347 (100.0)	340 (100.0)	141 (100.0)	51 (100.0)
Discontinued therapy, *n* (%)	216 (16.0)	142 (41.8)	55 (39.0)	29 (56.9)
Refractory disease, *n* (%)	172 (12.8)	131 (38.5)	49 (34.8)	26 (51.0)
Disease relapse, *n* (%)	157 (11.7)	35 (10.3)	8 (5.7)	2 (3.9)

*Note*: Discontinued therapy was defined as physician‐stated premature discontinuation of therapy prior to completion as prescribed. Refractory disease was defined as physician‐stated advancement of disease not following a complete response after the initiation of a given LOT and prior to the subsequent line. Disease relapse was defined as physician‐stated advancement of disease following a complete response after the initiation of a given LOT and prior to the subsequent line.

The most common treatment regimens by LOT are presented in Table 3. For 1L treatment, 63.6% (*N* = 857/1347) of patients received R‐CHOP, followed by 5.35% (*N* = 72/1347) of patients receiving DA‐R‐EPOCH. Among 2L patients, 17.9% (*N* = 61/340) received SCT (59 autologous and two allogeneic) with various salvage regimens, 13.5% (*N* = 46/340) received R‐ICE and 10.3% (*N* = 35/340) received BR. In 2L, 1.5% (*N* = 5/340) of patients received CAR‐T therapy with or without supplemental regimen(s). Among 3L patients, 9.9% (*N* = 14/141) received BR and polatuzumab vedotin, 9.9% (*N* = 14/141) received CAR‐T therapy with or without supplemental regimen(s), and 9.9% (*N* = 14/141) received SCT with various salvage regimens. Finally, among 4L patients, 11.8% (*N* = 6/51) received CAR‐T with or without supplemental regimen(s).

**TABLE 3:** Treatment regimen(s) by LOT.

	*n* (%)
**1L**	***N* = 1347**
R‐CHOP	857 (63.62)
DA‐R‐EPOCH	72 (5.35)
R‐EPOCH	35 (2.60)
R‐hyper‐CVAD/R‐MA	34 (2.52)
R‐CVP	12 (0.89)
R‐CEOP (Etoposide)	11 (0.82)
Other treatment regimens	326 (24.2)
**2L**	***N* = 340**
SCT (+/− any supplemental regimen)	61 (17.94)
R‐ICE	46 (13.53)
Bendamustine and rituximab (BR)	35 (10.29)
R‐GDP	26 (7.65)
R‐CHOP	17 (5.00)
R‐GemOx	17 (5.00)
Lenalidomide and rituximab (R2)	9 (2.65)
Other treatment regimens	129 (37.94)
**3L**	***N* = 141**
BR and polatuzumab vedotin	14 (9.93)
SCT (+/− any supplemental regimen(s))	14 (9.93)
CAR‐T (+/− any supplemental regimen(s))	14 (9.93)
R‐GemOx	8 (5.67)
R‐GDP	7 (4.96)
R‐ICE	7 (4.96)
BR	5 (3.55)
R2	5 (3.55)
Other treatment regimens	67 (47.52)
**4L**	***N* = 51**
CAR‐T (+/− any supplemental regimen(s))	6 (11.76)
BR and polatuzumab vedotin	3 (5.88)
Lenalidomide and tafasitamab	3 (5.88)
BR	2 (3.92)
BR and polatuzumab vedotin, rituximab, and polatuzumab vedotin	2 (3.92)
R2	2 (3.92)
R‐GDP	2 (3.92)
R‐ICE	2 (3.92)
R2‐I	2 (3.92)
Other treatment regimens	27 (52.94)

*Note*: “Other treatment regimens” indicates all other antineoplastic therapies received in the given LOT at lower frequencies than the abovementioned most common therapies. “Supplemental regimens” indicates conditioning, lymphodepletion, bridging, or other regimens.

Abbreviations: 1L, first line; 2L, second line; 3L, third line; 4L, fourth line; LOT, line of therapy; R‐CHOP, rituximab, cyclophosphamide, doxorubicin, vincristine, and prednisone.

**FIGURE 2:**
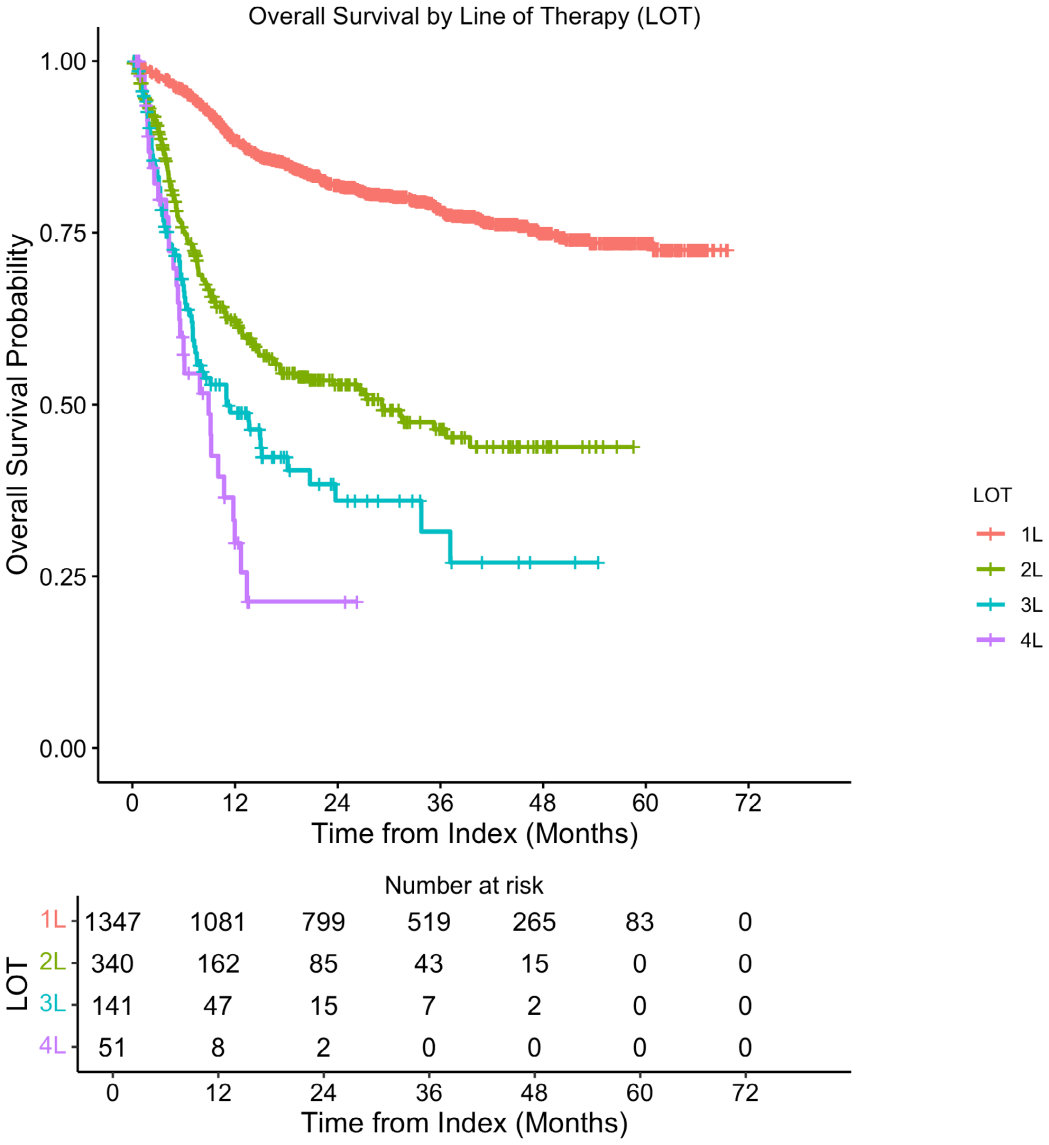
Overall survival by line of therapy.

**FIGURE 3:**
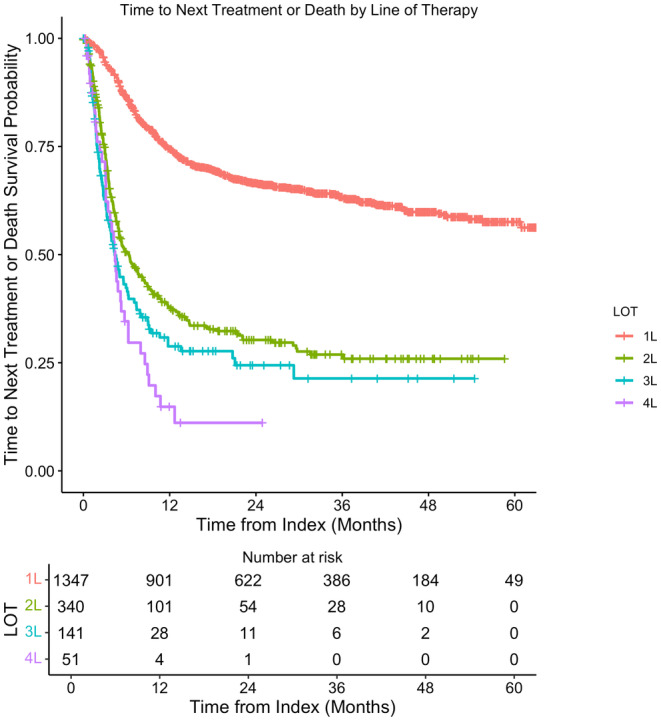
Time to next treatment or death by line of therapy.

The median rwTTNT was not reached (NR) for the 1L population. The median rwTTNT for the 2L, 3L, and 4L populations were 6.3 months (95% CI: 5.0, 8.5), 4.3 months (95% CI: 3.6, 6.3), and 4.4 months (95% CI: 3.1, 6.3), respectively (Figure 2 **).** Median follow‐up time from diagnosis and from 1L initiation among the study population was 28.0 (IQR: 14.6, 43.0) and 26.7 months (IQR: 13.6, 41.9), respectively. Median rwOS time was NR for the 1L population. The median rwOS times were 29.2 months (95% CI: 17.3, NR) for 2L, 11.0 months (95% CI: 7.4, 20.8) for 3L, and 8.9 months (95% CI: 5.5, 12.0) for 4L. One‐, 3‐, and 5‐year rwOS rates were 88.5% (95% CI: 86.8, 90.3), 78.4% (95% CI: 76.0, 80.9), and 73.5% (95% CI: 70.5, 76.7), respectively, from the start of 1L therapy. One‐ and 3‐year rwOS rates were 62.4% (95% CI: 57.1, 68.1) and 46.4% (95% CI: 40.1, 53.7) for 2L and 48.8% (95% CI: 40.4, 58.9) and 31.5% (95% CI: 21.3, 46.6) for 3L treated patients, respectively. The 1‐year rwOS rate among patients who initiated 4L was 29.8% (95% CI: 17.9, 49.8). The KM curves illustrate these findings (Figure 3).

## 4 DISCUSSION

This study provides contemporary data on patient characteristics, treatment patterns, and outcomes among patients treated for DLBCL in the real‐world setting. Over a median follow‐up time of 26.7 months from 1L initiation, 25.2% of 1L treated patients went on to receive 2L therapy. Furthermore, a numerically higher proportion of patients who had 2L therapy proceeded to receive subsequent LOTs (41.5% of 2L patients received 3L and 36.2% of 3L patients received 4L) compared to the 25.2% of 1L patients who proceeded to receive 2L therapy. Our study also documented remarkable rwOS disadvantages and disproportionately higher premature treatment discontinuations among patients with DLBCL who received later LOTs. While R‐CHOP remained the most common standard therapy in the 1L setting, regimens in later LOTs were heterogenous.

In our study, the key demographic and clinical characteristics, including age, race, and sex, were broadly consistent with the literature.^7^ Due to existing evidence of differential outcomes between patients with GCB and ABC subtypes, we examined the percentages of patients with each subtype (about 39% GCB, 32% ABC, 0.4% both subtypes, and 28% unknown) and found the proportions to be comparable to the literature, including Lenz et al. citing about 46% GCB, 40% ABC, and 14% unclassified.^12^ Furthermore, there were no major differences in proportions of each subtype among patients proceeding to later LOTs, although subtype missingness (28% in the overall population) could affect our finding.

Previous studies have demonstrated the widespread utilization of R‐CHOP in 1L for patients with DLBCL, similar to that shown in our study.^4^, ^13^ In this analysis, about 25% of patients who received 1L went on to receive 2L therapy over a median follow‐up time of 26.7 months from 1L therapy. Comparable studies, including Yang et al. and Danese et al., showed similar proportions of 1L patients who went on to receive 2L therapy (23% of incident patients and 20% of 1L patients, respectively), while Morrison et al. showed only 11.4% continued on to 2L, which could be due to differences in inclusion and exclusion criteria (e.g., qualified 1L treatments and treatment windows, and enrollment period) or follow‐up time (22.7 months from diagnosis date or 20.6 months from treatment start).^4^, ^7^, ^14^ Of patients who received 2L therapy in our study, 41.5% received 3L therapy. In studies by Klink et al., Morrison et al., and Yang et al., 65.6%, 14.6%, and 21.9% of patients who received 2L went on to receive 3L respectively.^4^, ^7^, ^15^ Of note, our study used more recent study data, and these studies leveraged different types of data sources and study populations.

Among patients who receive later LOTs, standard therapies are less defined. In our study, 17.4% (*N* = 59/340) of 2L patients received autologous SCT with various salvage regimens, 13.5% (*N* = 46/340) received R‐ICE, and 10.3% (*N* = 35/340) received BR. These estimates are consistent with evidence reported in previous studies.^7^, ^15^, ^16^ These treatment patterns highlighted notably lower uptake of therapies in later lines of treatment, including CAR‐T (9.9% in 3L and 11.8% in 4L). Limited uptake of SCT and CAR‐T in R/R DLBCL may be due to a number of factors including critical gaps in access to these newer and more effective treatments.^17^, ^18^ Previous studies have noted that some patients will not receive SCT due to issues with tolerability (i.e., advanced age) or access (i.e., distance to transplant facility and racial and socioeconomic disparities), and up to half of patients who are eligible for SCT will not receive it due to ineffective salvage treatment.^15^, ^19^, ^20^, ^21^ Similarly, patients who could receive treatment with CAR‐T therapy will also not receive treatment due to access barriers, referral challenges, lack of response to bridging therapy, or other factors.^18^, ^22^

Our results also showed that a large proportion of patients who received 2L therapy went on to receive later LOTs (41.5% received 3L and 36.2% of 3L patients received 4L). Notably, 23.0% of 1L patients (*N* = 310/1347) experienced a progression/relapse event within 12 months of 1L initiation. In other words, 94.2% of patients who experienced a progression/relapse event following 1L and prior to 2L initiation did so within 12 months of 1L initiation. This is comparable to results shown in Klink et al., where 85% of patients experienced early relapse.^15^ The higher proportions of patients who received later LOTs with refractory or early relapse disease indicate unmet need among these patients. Furthermore, our findings indicated poor rwOS and rwTTNT among patients who received later LOTs. This is consistent with the SCHOLAR‐1 study, which demonstrated that patients with R/R DLBCL experienced poor outcomes.^5^ These and other studies demonstrated that patients with R/R DLBCL experience suboptimal outcomes across later LOTs, highlighting the persistent need for additional, effective treatment options.^7^, ^16^ Accordingly, NCCN guidelines recommend clinical trials in instances where CAR‐T therapy is inaccessible or not indicated for treatment in later LOTs.^23^

One strength of our study is that we used the COTA oncology database with granular demographic and clinical information. The database includes patients treated at both academic centers and community practice networks with a primary geographic concentration in the Northeast and Southern regions of the United States. Although the geographic representation is limited to these primary regions, the provider site catchment areas extend beyond each primary region and represent diverse patient populations throughout the United States. Given the nature of COTA's data source and collection, these results are generalizable to appropriate patient populations, and patient characteristics, including sex, race, and age distribution, are comparable to those in published, population‐based data.^24^ Another strength is the contemporaneousness of the study population, which allowed for the analysis of current treatment patterns and outcomes in patients with DLBCL treated in the real‐world setting. Given that much of the existing literature focuses on patient populations from 2018 and earlier, our study, examining patients who initiated therapy and were diagnosed between January 2016 and March 2021, provides critical evidence on the more contemporary treatment landscape. We do, however, acknowledge the limitations related to the nature of real‐world data and the analytics applied to such datasets. Data collected from EHR sources may be subject to missingness as treatment provided and comorbidities treated outside of the provider network, or in an in‐patient setting, may not be fully captured. Similarly, lack of explicit documentation may result in missingness, as data abstraction relies on documentation of specific clinical variables. For example, although a patient may have been assessed for SCT or CAR‐T eligibility, in absence of a note describing the eligibility assessment, the patient will have missing data for these elements. This could result in potential misclassification of patient characteristics, treatments, or outcomes. Furthermore, diagnostic testing, clinical care, and outcome determinations may not be uniform in the real‐world setting. Additionally, mortality and adverse event data documentation in the real‐world setting does not adhere to the same reporting requirements as clinical trials, potentially resulting in missingness. COTA addresses potential gaps in mortality reporting by creating a composite mortality variable that is supplemented with commercially available obituary data. rwOS results in this study were comparable to those in the literature, supporting the use of this composite variable. Finally, algorithmically assigned LOT may not capture all nuances in real‐world clinical practice, leading to potential misclassification.

In conclusion, we found that a meaningful proportion (one‐quarter) of patients diagnosed with DLBCL who initiated 1L therapy proceeded to receive 2L therapy, indicating development of R/R disease to 1L over a median 26.7 months of follow‐up time from 1L initiation. The proportions of patients that received newer therapeutic options within the appropriate later LOTs were relatively low. Furthermore, we showed that patients with R/R disease who received 3L and 4L had striking rwOS disadvantages compared to patients who only received 1L. There is a high unmet need for novel and effective treatment options for patients with R/R DLBCL.

## AUTHOR CONTRIBUTIONS

**Helmneh M. Sineshaw:** Conceptualization (equal); methodology (equal); supervision (equal); writing – review and editing (equal). **Christina M. Zettler:** Methodology (equal); project administration (equal); writing – original draft (equal); writing – review and editing (equal). **Jennifer Prescott:** Conceptualization (equal); methodology (equal); supervision (equal); writing – review and editing (equal). **Mahek Garg:** Methodology (equal); writing – review and editing (equal). **Samhita Chakraborty:** Methodology (equal); writing – review and editing (equal). **Eric Sarpong:** Methodology (equal); writing – review and editing (equal). **Claire Bai:** Formal analysis (equal); methodology (equal); visualization (equal); writing – review and editing (equal). **Andrew J. Belli:** Methodology (equal); project administration (equal); writing – review and editing (equal). **Laura L. Fernandes:** Formal analysis (equal); methodology (equal); writing – review and editing (equal). **Ching‐Kun Wang:** Methodology (equal); supervision (equal); writing – review and editing (equal).

## FUNDING INFORMATION

Merck Sharp & Dohme LLC, a subsidiary of Merck & Co., Inc., Rahway, NJ, USA.

## CONFLICT OF INTEREST STATEMENT

Helmneh M. Sineshaw, Jennifer Prescott, Mahek Garg, and Eric M Sarpong report current employment and stock ownership and/or stock options in Merck & Co., Inc., Rahway, NJ, USA. Samhita Chakraborty reports prior employment and stock ownership and/or stock options in Merck & Co., Inc., Rahway, NJ, USA. Christina M. Zettler, Claire Bai, Andrew J. Belli, Laura L. Fernandes, and Ching‐Kun Wang report employment and equity in COTA, Inc., New York, NY, USA.

## Data Availability

The data underlying this article were provided by COTA, Inc. and cannot be shared due to privacy reasons. Summary‐level data are provided throughout the manuscript and in the accompanying tables and figures.
